# The Concentration of Carbon Source in the Medium Affects the Quality of Virus-Like Particles of Human Papillomavirus Type 16 Produced in *Saccharomyces cerevisiae*

**DOI:** 10.1371/journal.pone.0094467

**Published:** 2014-04-08

**Authors:** Hyoung Jin Kim, Yingji Jin, Hong-Jin Kim

**Affiliations:** Laboratory of Virology, College of Pharmacy, Chung-Ang University, Dongjak-Gu, Seoul, South Korea; Instituto Butantan, Brazil

## Abstract

There is accumulating evidence that virus-like particles (VLPs) recombinantly produced in *Saccharomyces cerevisiae* (*S. cerevisiae*) are characterized by low structural stability, and that this is associated with reduced antigenicity and immunogenicity. However, little attention has been devoted to methods of improving the quality of the VLPs. Here, we investigated the effect of carbon source concentration in the medium on the antigenicity and immunogenicity of human papillomavirus (HPV) type 16 L1 VLPs expressed in *S. cerevisiae* from the galactose promoter. Media containing 2, 4, 6, and 8% carbon source, composed of both glucose and galactose in equal proportion, were used. VLP antigenicity was enhanced in cultures grown on media with 6 or 8% carbon source, compared to those from cultures with less than 6% carbon source. Moreover, the VLPs obtained from these cultures induced higher anti-HPV16 L1 IgG titers and neutralizing antibody titers in immunized mice than those purified from cultures with less than 6% carbon source. Our results indicate that the concentration of the carbon source in the medium plays a crucial role in determining the antigenicity and immunogenicity of HPV type16 L1 VLPs.

## Introduction

The ongoing technological advances in genetic engineering and in the production of recombinant proteins have enabled the development of subunit vaccines that utilize as antigens monomeric proteins derived from pathogens [1]. Moreover, the development of peptide vaccines using synthetic pathogen epitopes has posed a considerable challenge [2]. Undoubtedly this challenge and others have contributed to the diversification of vaccine technologies and increased our understanding of mechanisms of infection. At the same time, however, a growing body of research suggests that both subunit and peptide vaccines have low immunogenicity, and that their ability to elicit neutralizing antibodies is quite limited [3], [4]. The low levels of pathogen-specific complexes and tertiary structures in these vaccines are regarded as the most problematic factors reducing their utility.

Virus-like particles (VLPs) are multimeric protein complexes similar in shape to naturally occurring virions [5], [6]. They are noninfectious and safer than conventional inactivated or attenuated vaccines because they do not contain viral genetic material [7]. However, the most significant advantage of VLPs is that they possess capsid-specific neutralizing epitopes and a highly ordered structure resulting from the assembly of their subunit proteins [8], [9]. These repetitive conformational epitopes on the surface of the VLPs stimulate antigen-presenting cells (APCs) more strongly than monomeric or disassembled antigens [8]. Moreover, due to their shape and size (20 – 100 nm), these particles are preferentially taken up by APCs [9]. Consequently VLPs appear to have a greater ability to stimulate the immune system and evoke protective immunity than subunit or peptide vaccines.

The recently developed strategy for producing VLPs has provided new insights into mechanisms protecting against pathogens, and yielded an innovative platform for developing high-efficacy vaccines. However, obtaining high-quality VLPs presents a challenge because of their structural complexity. *Saccharomyces cerevisiae* (*S. cerevisiae*) is one of the preferred expression systems used for producing VLPs [10]. It has been used to produce VLPs of the human hepatitis B virus (HBV), and the human papillomavirus (HPV) [10], [11], [12]; the HPV L1 recombinant proteins produced in *S. cerevisiae* possess the ability to self-assemble into VLPs. Another advantage of the yeast expression system is its low production cost. However, VLPs produced in this system are reported to have low structural stability [5], and this effect was correlated with decreased antigenicity and immunogenicity [13], [14].

Many *in vitro* strategies for increasing VLP antigenicity and immunogenicity, such as redox refolding, VLP maturation, and salt treatment, have been tried with some success [13], [15], [16]. However, insufficient attention has been paid to strategies for improving the quality of the VLPs during cell culture, despite the fact that VLP bioprocessing *in vivo* relies on intracellular assembly. In this study, we compared the antigenicity and immunogenicity of HPV type 16 L1 protein (HPV16 L1) VLPs produced in the *S. cerevisiae* expression system with different concentrations of carbon source. Previously, we found that the concentration and composition of the carbon source used for *S. cerevisiae* producing HPV16 L1 protein significantly affect the yield of the HPV16 L1 protein [17]. In this study, we report that the concentration of the carbon source in yeast cultures substantially affects the quality of HPV16 L1 VLPs.

## Materials and Methods

### Ethics

All animal experiments were treated in accordance with the guideline of Institutional Animal Care and Use Committee, Chung-Ang University IACUC, and the protocol was approved by the IACUC. The conditions of mice were monitored twice a day. Mice were anesthetized intraperitoneally with 10 μl of 4∶1 mixture of Zoletil 50 (Virbac, France) and Rompun (Bayer Animal Health, Germany) prior to blood collection.

### Production of HPV16 L1 VLPs

The codon-optimized HPV16 L1 gene (HPV16 L1 gene-opt), designed to reduce the secondary structure of the mRNA [18], was ligated into YEGα-MCS vector. *S. cerevisiae* Y2805 was transformed with the resulting plasmid (YEGα-MCS-HP16 L1 gene-opt). Transformants were selected on SD-ura medium, a synthetic medium without uracil, and inoculated into 150 mL of YPDG medium. The YPDG medium contained 1% yeast extract, 2% peptone, and various concentrations of carbon source. For the purpose of this study, the selected concentrations were 2, 4, 6 and 8%, and the glucose to galactose ratio was set at 1∶1. Therefore, the percentages of glucose and galactose in the media were 1%, 2%, 3% and 4% each. All components used to prepare media were purchased from Duchefa (Netherlands). Cells were cultured for 6 days at 30°C with shaking at 230 rpm.

### Purification of HPV16 L1 VLPs

HPV16 L1 protein was purified as previously described [19]. Briefly, the cells were disrupted with glass beads using a vortex mixer (BioSpec Products, USA), and the cell lysate and beads were removed by centrifugation at 14000×*g* for 10 min. Thereafter L1 protein was recovered from the cell lysate as pellet by precipitation with 40% saturated ammonium sulfate. The pellet was resuspended in the buffer (10 mM sodium phosphate buffer, pH 7.2, containing 0.15 M NaCl, and 0.01% Tween 80), and dialyzed against the same buffer for 4 h at room temperature (RT). After dialysis precipitation of contaminating proteins was induced in 10 mM sodium phosphate buffer (pH = 7.2, containing 0.15 M NaCl, and 0.01% Tween 80) for 16 h at 4°C, and the precipitates were removed by centrifugation at 12000×*g* for 10 min. The supernatant (fraction containing L1 protein) was dialyzed against binding buffer for cation-exchange chromatography (2.68 mM KCl, 1.47 mM KH_2_PO_4_, 8.1 mM Na_2_HPO_4_, 0.5 M NaCl, pH 7.2+0.01% Tween 80) for 3 h at RT and loaded onto a column packed with P-11 phosphocellulose cation-exchange resin (1.8×3 cm, 3 ml of resin, Whatman, UK). The column was washed with five column volumes of the binding buffer, and bound proteins were eluted by successive addition of buffer containing 0.6, 0.7, 0.8, 0.9 and 1 M NaCl. L1 protein fractions were collected and concentrated using an Amicon Ultra-4 (Millipore, USA) and dialyzed against storage buffer (pH = 7.2, 2.68 mM KCl, 1.47 mM KH_2_PO_4_, 8.1 mM Na_2_HPO_4_, 0.325 M NaCl and 0.01% Tween 80).

### Measuring protein concentrations

Protein concentrations were measured by the Bradford protein assay (Bio-Rad Laboratories, USA) with bovine serum albumin (BSA; Pierce, USA) as a standard. The purity and quantity of L1 were determined by SDS-PAGE and Western blot analysis.

### SDS-PAGE and Western blot analysis

SDS-PAGE and Western blotting were performed as described [17]. Tubulin was detected using rat anti-tubulin antibody (Abcam, USA). Band intensities corresponding to L1 were determined with Image J software (http://rsbweb.nih.gov/ij/).

### Transmission electron microscopy (TEM)

Electron microscopy was performed on a TEM200CX at a final magnification of 234000×. The purified HPV16 L1 VLPs were absorbed onto carbon-coated grids, stained with 2% phosphotungstic acid and analyzed under the microscope [20].

### Enzyme-linked immunosorbent assay (ELISA) to detect neutralizing epitopes on HPV16 L1 VLPs

The ELISA was carried out as described [13]. Briefly, a 96-well ELISA plate (Greiner Bio One, Germany) was coated overnight with 400 ng of purified HPV16 L1 VLPs per well and blocked with 5% skim milk in PBS containing 0.05% Tween 20 (PBST). The plate was incubated with 250 ng/mL of anti-HPV16 neutralizing monoclonal antibodies (Mabs), H16.V5 or H16.E70, for 1 h at 37°C. The Mabs bound to VLPs were detected using HRP-conjugated goat anti-mouse IgG antibody (Bethyl Laboratories, USA). Color reactions were developed with *o*-phenylenediamine (Sigma, USA), and optical density was measured at an absorbance of 492 nm.

### Dynamic light scattering (DLS)

DLS was performed using an ELSZ-2 system (Otsuka Electronics, Japan) as described [21].

### Mice immunization

Six-week-old female BALB/c mice (Orient Bio, South Korea) were immunized three times subcutaneously with purified HPV16 L1 VLPs at two-weekly intervals. The mice were divided into five groups of 8, 9, or 10 mice each. The control group received PBS, and the remaining groups received HPV16 L1 VLPs purified from cultures containing 2, 4, 6, or 8% carbon source. The mice received 10 or 1000 ng purified HPV16 L1 VLPs per dose in combination with aluminum hydroxide (200 μg per dose). The doses were as recommended in a previous report [13]. Mouse sera were obtained from tail veins ten days after the third immunization.

### Titration of anti-HPV16 L1 IgG

The anti-HPV16 L1 IgG titers of mouse sera were measured as described [13], [14]. Briefly, 96-well ELISA plates were coated overnight with 100 ng of purified HPV16 L1 VLPs per well and blocked with 5% skim milk in PBST. The plates were reacted with three- or four-fold serial dilutions of mouse sera for 1 h at 37°C. The anti-HPV16 L1 IgG bound to the coated HPV16 L1 VLPs was detected using HRP-conjugated goat anti-mouse IgG antibody (Bethyl), and color reactions were developed as described above.

### Neutralization assays using HPV16 pseudovirus (HPV16 PsV)

HPV16 PsV was prepared according to a published method [16]. Neutralizing activity in mouse sera was determined as described [8]. The equation used was as follows: neutralization (%)  =  (value for PsV alone – value for PsV + mice serum)/(value for PsV alone – value for blank) × 100.

The neutralizing antibody titer of a mouse serum was defined as the reciprocal of the highest dilution that caused a reduction of at least 50% in secreted embryonic alkaline phosphatase (SEAP) activity [22].

## Results

### Final quantities and purities of HPV16 L1 VLPs

**Figure 1:**
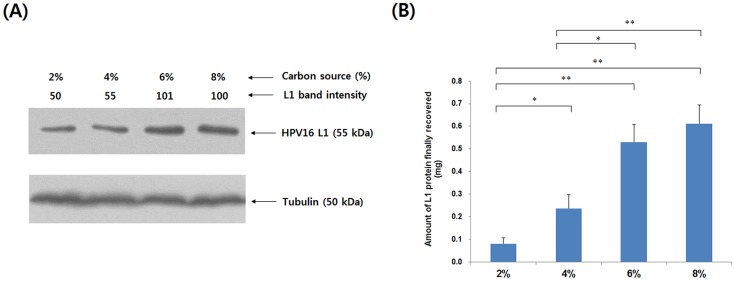
Production and purification of HPV16 L1 VLPs. (A) Quantities of L1 protein in cell lysates were compared by Western blotting. (2 μg of cell lysate protein were loaded per well). Tubulin was used as internal control. Band intensities were determined as described in Materials and methods. (B) Quanties of L1 VLPs finally recovered after purification. Cells were cultured in 150 mL YPDG medium for 144 h. 2, 4, 6, and 8% indicate the concentrations of total carbon source in YPDG medium. *p<0.05; **p<0.01. Values represent the mean ± SEM of six independent purifications.

Production of HPV16 L1 protein was evaluated as a function of carbon source concentration (Fig. 1A), and the quantities of L1 recovered after purification were compared between groups (Fig. 1B). Fig 1A illustrates that the specific L1 protein production (mg L1/mg protein in lysate) increases with increasing carbon source concentration. Fig 1B is a graph showing the amount of L1 protein finally recovered after purification, which would be expected to increase as cell yield increases with cell mass in culture. These results indicate that the enhanced production of L1 protein in culture with 6% or 8% carbon source was reflected in the final yields of L1 protein (Fig. 1B). The amount of L1 protein obtained from the culture containing 8% carbon source was, respectively, 7.6-, 2.5-, and 1.2-fold higher than the amount obtained from the 2, 4, and 6% cultures.

**Figure 2:**
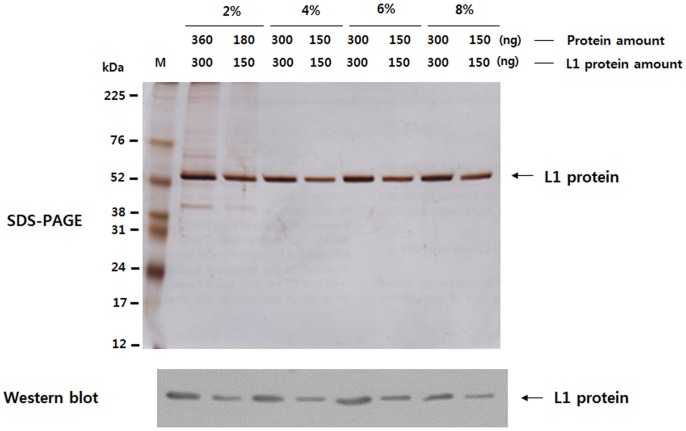
SDS-PAGE and Western blotting of purified HPV16 L1 VLPs. 360 or 180-2%, and 300 or 150 ng of protein was loaded per well for the other VLPs. L1 protein was visulized by silver staining and Western blotting. M indicates the molecular weight marker.

We confirmed that the L1 protein finally recovered was able to form VLPs (data not shown). The concentration of L1 in each preparation was estimated by SDS-PAGE and Western blotting (Fig. 2) prior to analysis by ELISA and DLS, and the immunization of mice. The HPV16 L1 VLPs obtained from cultures containing 2, 4, 6, and 8% carbon source are referred to as HPV16 L1 VLPs-2%, HPV16 L1 VLPs-4%, HPV16 L1 VLPs-6% and HPV16 L1 VLPs-8%, respectively. As shown in Fig. 2, the HPV16 L1 VLPs-2% preparation was less pure than the other preparations: the proportion of L1 in the total protein was estimated to be 80%. Because of this, a 1.2-fold greater amount of HPV16 L1 VLPs-2% was used in the subsequent analyses.

### Comparison of *in vitro* antigenicity of the HPV16 L1 VLPs

**Figure 3:**
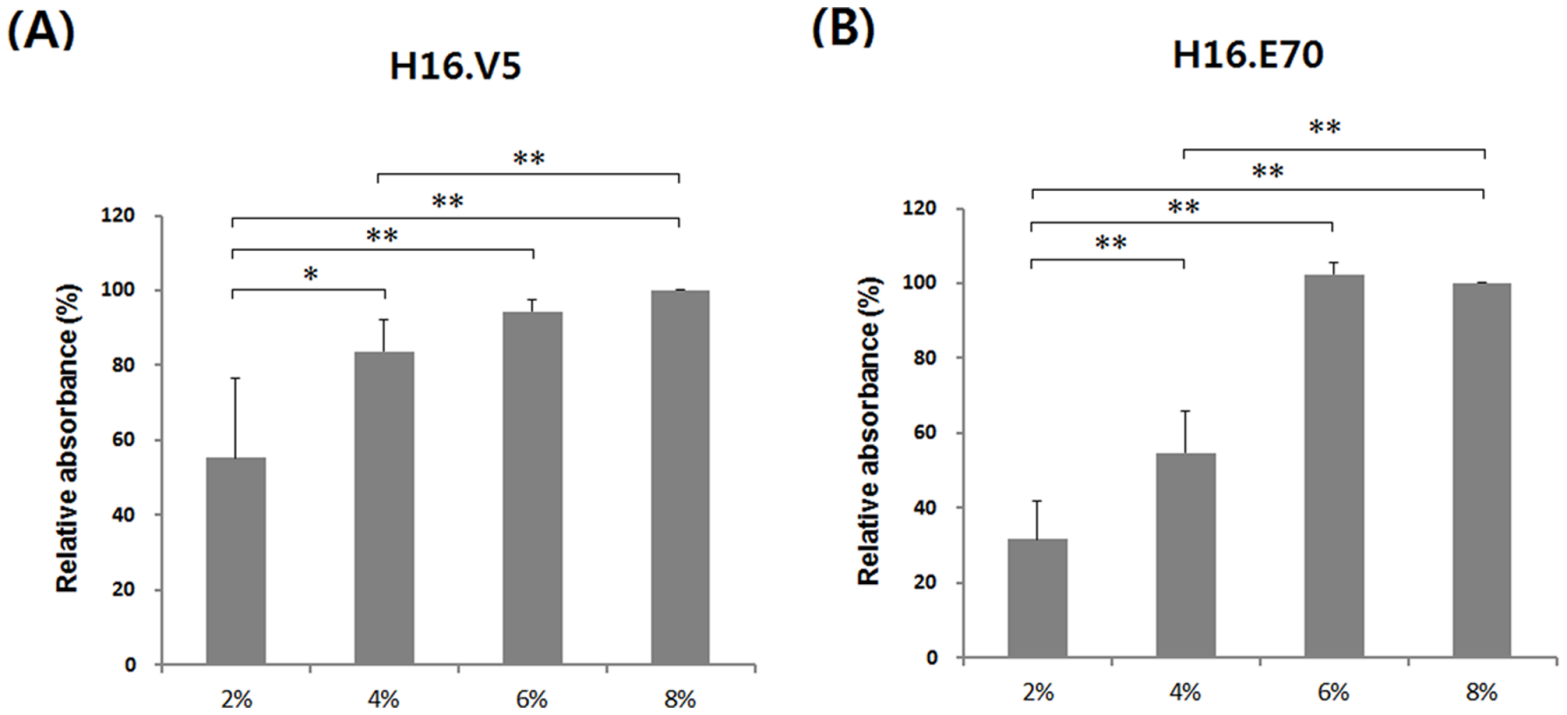
Reacitivity of HPV16 L1 VLPs towards H16.V5 and H16.E70. Reactivity of Mabs towards HPV16 L1 VLPs was determined as described in Materials and methods. Panels A and B show the reactivity of each type of VLP towards H16.V5 and H16.E70, respectively. The absorbance of HPV16 L1 VLP-8% was set at 100%. Values represent the mean ± SEM of four independent experiments. *p<0.05; **p<0.01.

To investigate the neutralizing epitopes in each VLP preparation, ELISAs were performed using neutralizing Mabs, H16.V5 and H16.E70 (Fig. 3). It is suggested that H16.V5 recognizes neutralizing epitopes located in the FG and HI loops, while H16.E70 recognizes those in the FG loop [23]. As shown in Fig. 3, HPV16 L1 VLPs-6% and -8% had higher reactivity against H16.V5 and H16.E70 than HPV16 L1 VLPs-2% and -4%. It has been suggested that the hydrodynamic diameter of VLPs is inversely related to their antigenicity and immunogenicity [14], [24]. Figure S1 shows DLS plots for the four types of HPV16 L1 VLPs. The hydrodynamic sizes of the VLPs from cultures with 6% and 8% carbon source were smaller than in the remaining cultures. These results support that adding 6% or 8% of carbon source to culture improves the antigenicity of the resulting HPV16 L1 VLPs.

### Antibody response following immunization with HPV16 L1 VLPs

**Figure 4:**
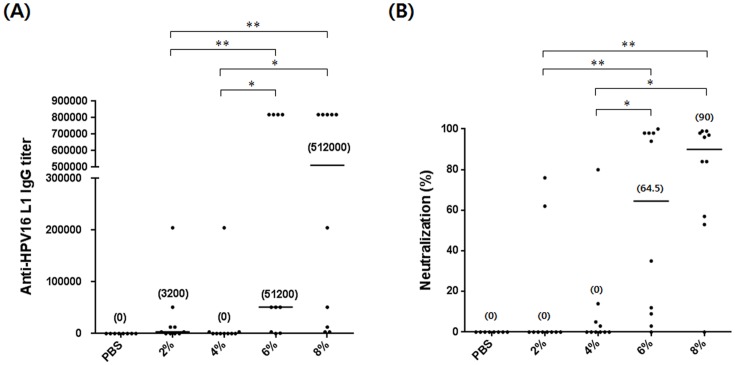
Antibody responses following three immunizations with 10 ng of HPV16 L1 VLPs per dose. (A) Anti-HPV16 L1 IgG titers and (B) neutralizing activities against HPV16 PsVs in mouse sera immunized with HPV16 L1 VLPs. Median values in (A) and (B) are in parentheses. *p<0.05; **p<0.01. PBS, n = 8; 2%, n = 10; 4%, n = 10; 6%, n = 10; 8%, n = 10.

**Figure 5:**
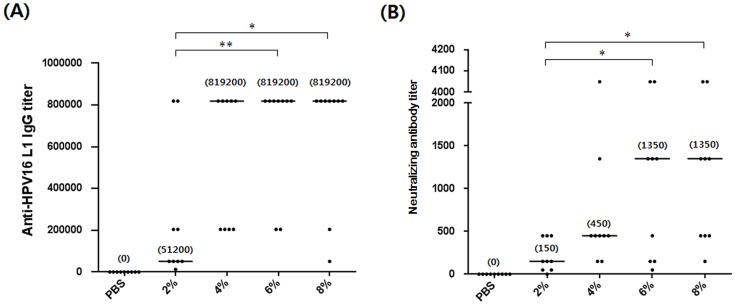
Antibody responses following three immunizations with 1000 ng of HPV16 L1 VLPs per dose. (A) Anti-HPV16 L1 IgG titers and (B) neutralizing antibody titers against HPV16 PsVs in mouse sera immunized with HPV16 L1 VLPs. Median values in (A) and (B) are in parentheses. PBS, n = 9; 2%, n = 9; 4%, n = 9; 6%, n = 9; 8%, n = 9.

Figures 4 and 5 show the antibody responses in mice immunized three times with 10 or 1000 ng of the HPV16 L1 VLPs per dose, respectively. HPV16 L1 VLPs-6% and -8% induced higher levels of anti-HPV16 L1 IgG titers and neutralizing activities against HPV16 PsVs than HPV16 L1 VLPs-2% and -4% in mice immunized with 10 ng of HPV16 L1 VLPs per dose (Fig. 4). The same was true when the mice received 1000 ng of HPV16 L1 VLPs per dose: the neutralizing antibody titers induced by HPV16 L1 VLPs-6% and -8% were 9 and 3 times higher than those induced by HPV16 L1 VLPs-2% and -4%, respectively (Fig. 5B). Although the median anti-HPV16 L1 IgG titer induced by HPV16 L1 VLPs-4% was the same as that induced by HPV16 L1 VLP-6% and -8% (Fig. 5A), the median neutralizing antibody titer was significantly lower than those induced by HPV16 L1 VLPs-6% and-8% (Fig. 5B). These results support the finding that yeast culture containing 6% or 8% of the carbon source offers HPV16 L1 VLPs superior antigenicity and immunogenicity.

## Discussion

Typically, cultures designed to produce heterologous proteins in *S. cerevisiae* contain 2 – 4% carbon source due to the fact that higher concentrations can cause osmotic stress to the yeast and lead to higher alcohol production, which is potentially detrimental to cultures [25], [26]. Under these typical conditions, the preferred culture period is 48–72 h, because longer culture times can exhaust the energy source.

In contrast with conventional fermentation strategies, in this study we obtained superior-quality HPV16 L1 VLPs from cultures containing 6 and 8% carbon source and after 6 days of culture. As shown in Fig. 1A, the yields of L1 protein increased with increasing concentrations of carbon source. Higher concentrations of L1 were previously found to facilitate the assembly of L1 proteins *in vitro* [27]. Therefore, the high L1 levels derived from the 6 or 8% carbon source cultures in our study may provide a favorable environment for the assembly of L1 into VLPs during cell culture and purification.

Previously we found that the levels of conformational neutralizing epitopes on the surface of HPV16 VLPs increased with increasing culture duration when the culture contained more than 6% carbon source [17]. In the same study, we found that the proportion of soluble L1 protein in culture increased with longer culture duration [17]. These results demonstrated that in the yeast expression system longer culture periods may be necessary to obtain correctly folded L1 proteins that go on to form highly immunogenic HPV L1 VLPs. Based on our present findings, it appears that higher concentrations of carbon source make it possible to increase culture duration (due to the long stationary phase), which provides an opportunity for the yeast cells to make immunogenic L1 protein.

Accumulating evidence suggests that the redox status of host cells plays a pivotal role in the infection and replication of viruses [28], [29]. Also, *in vitro* treatment of redox reagent affects VLP conformation and maturation [16]. Reduced glutathione (GSH) acts as a radical scavenger, with its redox-active sulfhydryl group reacting with oxidants to produce oxidized glutathione (GSSG) [30]. We have found that the levels of GSSG decreased in favor of GSH in prolonged cultures of *S. cerevisiae* producing HPV L1 (Figure S2), indicating that the intracellular redox state undergoes considerable change during the cell culture. The GSSG and GSH were measured as described previously [31]. Therefore, it is possible that a correlation exists between the folding and assembly of HPV L1 and intracellular redox status.

Various culture conditions have been tested in an attempt to produce VLPs in the yeast expression system. Similarly, various types of purification protocols have been developed for VLPs. In previous reports we suggested that the choice of purification method could affect the structural integrity and immunogenicity of HPV16 L1 VLPs [13], [14]. Recently, Xie *et al*. suggested that column-purified VLPs have superior structural integrity to those purified by cesium chloride ultracentrifugation, a traditional purification method for viruses [32]. Our present results appear to confirm that culture conditions affect the quality of HPV16 L1 VLPs. Moreover, they suggest that the properties of VLPs are influenced by their *in vivo* and *in vitro* environments, indicating that much effort should be devoted to production protocols in order to achieve superior-quality VLPs. In the light of these findings, more diversified criteria for monitoring the quality of VLPs may be required. It is likely that further study of the changes in immunogenicity and structural integrity of VLPs as a function of culture conditions will provide valuable insights and help to develop high-efficacy and low-cost VLP vaccines.

## Supporting Information

Figure S1 **DLS analysis of purified HPV16 L1 VLPs.** (A) Representative plot of all VLPs: numbers in parentheses indicate the hydrodynamic diameters of the HPV16 L1 VLPs. (B) The mean ± SEM of four independent experiments.
(TIF)

Figure S2 **GSSG and GSH level of** ***S. cerevisiae*** **producing HPV16 L1 protein.** Cells were culture in YPDG medium containing 7% glucose and 1% galactose for 144 h at 30°C. This culture condition showed the highest production yield of the L1 protein (see reference [17]). The intracellular levels of GSSG and GSH were determined as described [31] with modification. Cells were disrupted by vortex with glass beads, and cell debris was removed by centrifugation. The protein concentrations of the cell lysates were determined by Bradford protein assay and adjusted to 1 mg/ml. Deproteinization was performed by addition of sulfosalicylic acid (final concentration of sulfosalicylic acid: 3%). Thereafter, the GSSG and GSH levels of the deproteinized lysates were measured. A and B shows cell density and L1 protein production. Cell density was measured at 600 nm, and L1 protein was detected by Western blotting. C and D are results measuring intracellular GSSG and GSH level, respectively. Data are mean ± SD of duplicate assays. The GSSG and GSH level of cells cultured for 24 h were set at 100%, respectively.
(TIF)
