# Supplementary figures and images for: The Concentration of Carbon Source in the Medium Affects the Quality of Virus-Like Particles of Human Papillomavirus Type 16 Produced in Saccharomyces cerevisiae
Source: PLoS One. 2014 Apr 8;9(4):e94467. doi: 10.1371/journal.pone.0094467 (PMC3979840; doi:10.1371/journal.pone.0094467)

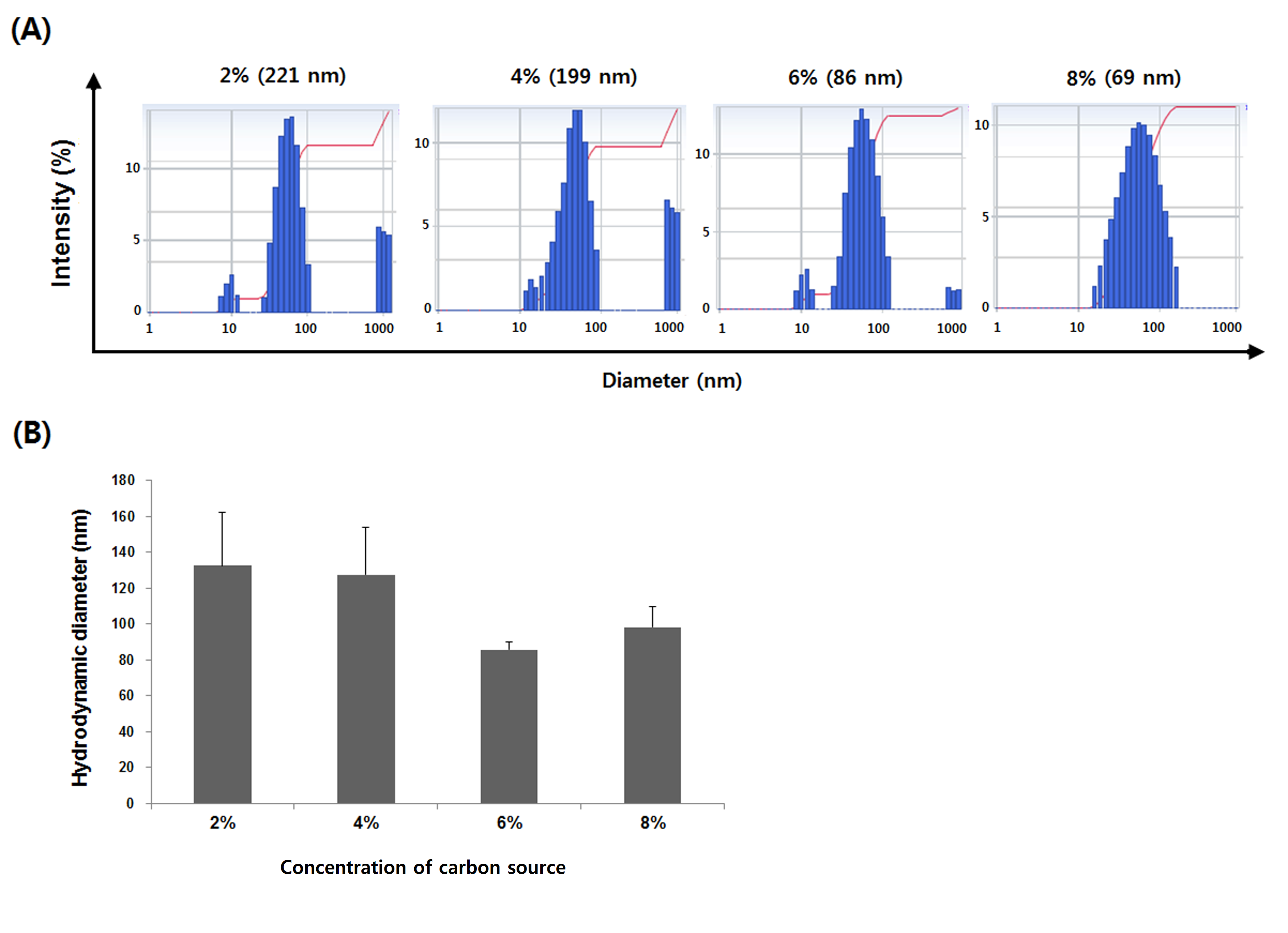

Supplement: Figure S1 — DLS analysis of purified HPV16 L1 VLPs. (A) Representative plot of all VLPs: numbers in parentheses indicate the hydrodynamic diameters of the HPV16 L1 VLPs. (B) The mean ± SEM of four independent experiments. (TIF) [file pone.0094467.s001.tif]

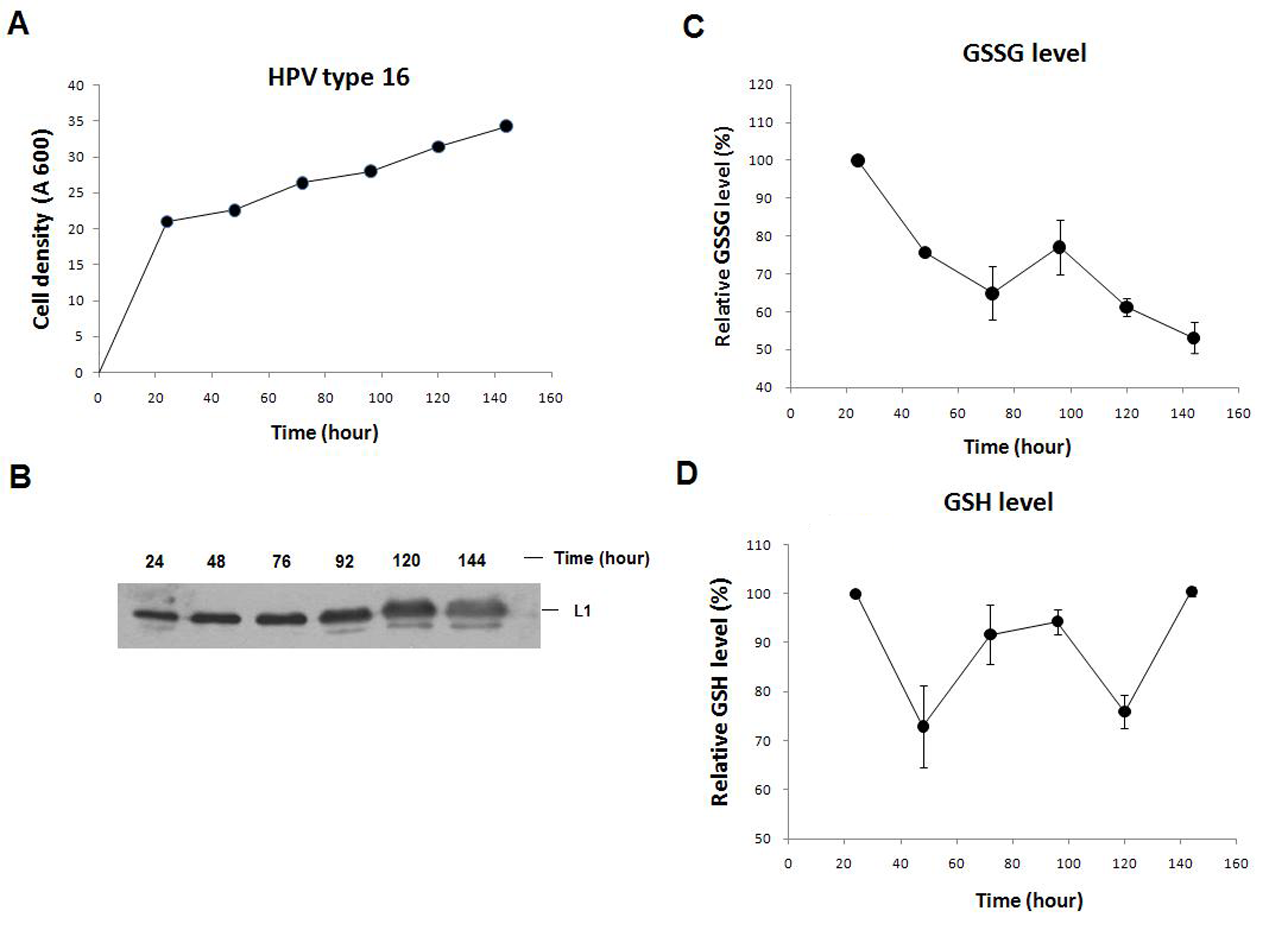

Supplement: Figure S2 — GSSG and GSH level of S. cerevisiae producing HPV16 L1 protein. Cells were culture in YPDG medium containing 7% glucose and 1% galactose for 144 h at 30°C. This culture condition showed the highest production yield of the L1 protein (see reference [17]). The intracellular levels of GSSG and GSH were determined as described [31] with modification. Cells were disrupted by vortex with glass beads, and cell debris was removed by centrifugation. The protein concentrations of the cell lysates were determined by Bradford protein assay and adjusted to 1 mg/ml. Deproteinization was performed by addition of sulfosalicylic acid (final concentration of sulfosalicylic acid: 3%). Thereafter, the GSSG and GSH levels of the deproteinized lysates were measured. A and B shows cell density and L1 protein production. Cell density was measured at 600 nm, and L1 protein was detected by Western blotting. C and D are results measuring intracellular GSSG and GSH level, respectively. Data are mean ± SD of duplicate assays. The GSSG and GSH level of cells cultured for 24 h were set at 100%, respectively. (TIF) [file pone.0094467.s002.tif]
